# New Detection Methods for Cryphonectria Hypovirus 1 (CHV1) through SYBR Green-Based Real-Time PCR and Loop-Mediated Isothermal Amplification (LAMP)

**DOI:** 10.3390/v16081203

**Published:** 2024-07-26

**Authors:** Ali Çelik, Deniz Çakar, Sibel Derviş, Ali Ferhan Morca, Seçil Akıllı Şimşek, Pedro Romon-Ochoa, Göksel Özer

**Affiliations:** 1 Department of Plant Protection, Faculty of Agriculture, Bolu Abant İzzet Baysal University, Bolu 14030, Türkiye; 2 Central Research Laboratory Application and Research Center, Çankırı Karatekin University, Çankırı 18100, Türkiye; 3 Department of Plant Protection, Faculty of Kızıltepe Agricultural Sciences and Technologies, Mardin Artuklu University, Mardin 47000, Türkiye; 4 Department of Plant and Animal Production, Vocational School of Kızıltepe, Mardin Artuklu University, Mardin 47000, Türkiye; 5 Directorate of Plant Protection Central Research Institute, Gayret Mah. Fatih Sultan Mehmet Bulv., Yenimahalle, Ankara 06172, Türkiye; 6 Department of Biology, Faculty of Sciences, Çankırı Karatekin University, Çankırı 18100, Türkiye; 7 Forest Research, Plant Pathology Department, Alice Holt Research Station, Farnham GU10 4LH, UK

**Keywords:** CHV1, colorimetric, LAMP, SYBR Green, qPCR

## Abstract

Some mycoviruses can be considered as effective biocontrol agents, mitigating the impact of phytopathogenic fungi and consequently reducing disease outbreaks while promoting plant health. *Cryphonectria parasitica*, the causal agent of chestnut blight and a highly destructive pathogen, experienced a notable decrease in its virulence with the identification of cryphonectria hypovirus 1 (CHV1), a naturally occurring biocontrol agent. In this study, two innovative diagnostic protocols designed for the accurate and efficient detection of CHV1 are introduced. The ORF A and ORF B regions of CHV1 are targeted by these techniques, which employ colorimetric loop-mediated isothermal amplification (LAMP) with 2 Colorimetric LAMP Master Mix and real-time quantitative PCR (qPCR) with SYBR Green chemistry, respectively. The LAMP assay presents a discernible color transition, changing from pink to yellow after a 35 min incubation period. Comparative analysis, when assessed against two established reverse transcription-PCR (RT-PCR) techniques, reveals a significant enhancement in sensitivity for both the LAMP approach, which offers a tenfold increase, and the qPCR method, which showcases a remarkable 100-fold sensitivity improvement. Throughout the comparison phase, it was evident that the RT-PCR, LAMP, and qPCR procedures displayed superior performance compared to the Bavendamm test, relying on phenol oxidase activity, effectively distinguishing hypovirulent strains. Consequently, this study introduces two pioneer diagnostic assays for highly sensitive CHV1 detection, representing a substantial advancement in the realm of CHV1 surveillance techniques. These methodologies hold significant promise for enhancing research endeavors in the domain of the biological control of *C*. *parasitica*.

## 1. Introduction

Mycoviruses harbor the potential to serve as effective biological control agents, eliciting suppressive effects on the virulence of phytopathogenic fungi. This influence ultimately leads to a decreased incidence of disease outbreaks and promotes the enhanced survival of host plants [1]. Fungal viruses are systematically categorized into discrete families based on their genetic attributes, encompassing double-stranded RNA (dsRNA) families, single-stranded RNA (ssRNA) families, reverse-transcribing virus families, and the single-stranded DNA (ssDNA) family, classified by the International Committee on Taxonomy of Viruses [2]. Some of these viral entities inflict detrimental effects on their fungal hosts, resulting in reduced growth, altered pigmentation, impaired conidiation (the formation of asexual spores), and diminished virulence. Some examples of fungal genera that experience these biological effects include *Cryphonectria* [3], *Diaporthe* [4], *Fusarium* [5], and *Rosellinia* [6].

Of particular significance, *Cryphonectria parasitica* (Murrill) M. E. Barr, commonly known as the causal agent of chestnut blight, inflicts its impact on *Castanea* species. The fungal pathogen, which originated in Eastern Asia according to Liu et al. [7], was introduced to North America in the late nineteenth century. Its introduction had a significant impact on the decrease and eventual extinction of *Castanea dentata* (Marshall) Borkh, as noted by Anagnostakis [8]. Subsequently, it has also affected sweet chestnut trees (*Castanea sativa* Mill.) across much of continental Europe, with its initial introduction occurring in Italy in 1938 [9]. The prevalence of *C. parasitica* has been noted by Çakır et al. [10] among all surveyed chestnut-growing areas in Türkiye. The aggressive epidemic caused by *C*. *parasitica*, despite its significant global destruction, had a notable decline with the identification of cryphonectria hypovirus 1 (CHV1), a naturally occurring biocontrol agent. To assess the pivotal role of CHV1 in promoting transmissible hypovirulence for the biological control of chestnut blight [11], CHV1 has undergone a thorough investigation, underscoring its substantial contribution to biocontrol initiatives [1].

CHV1 stands as the prototypical member of the *Hypoviridae* family [12,13]. *Hypoviridae* is a family of viruses lacking a capsid and characterized by positive-sense RNA genomes ranging from 7.3 to 18.3 kilobases. These genomes typically contain either a single large open reading frame (ORF) or two ORFs [13]. CHV1 possesses a dsRNA genome, a common feature among most fungal viruses. Notably, dsRNAs in isolation lack infectivity but can be horizontally transmitted via fungal anastomosis and vertically transmitted through asexual spores, with sexual spores not participating in this mode of transmission [14]. It is important to note that while hypovirus RNA is found as dsRNA in hyphal extracts, its structural features are similar to those observed in the replication processes of ssRNA viruses [15]. In the case of CHV1, its positive coding strand spans approximately 12.7 kilobases and is characterized by polyadenylation. This strand comprises two continuous open reading frames (ORFs A and B) encoding polyproteins, which undergo proteolytic processing [16].

Nucleic acid-based diagnostic techniques, as highlighted by McCartney et al. [17], play a pivotal role in early and specific pathogen detection. CHV1 diagnosis has historically relied on polymerase chain reaction (PCR) methods, with particular emphasis on the ORF A and ORF B genes [14,16,18,19,20,21,22,23,24]. Recent investigations have expanded beyond these areas, exploring probe-based real-time PCR [25,26,27] for enhanced CHV1 detection and quantification. Molecular techniques, including PCR and other nucleic acid-based diagnostic methods, have significantly advanced pathogen detection. However, their practicality is limited, especially in regions with resource constraints or inadequate access to advanced laboratory facilities [28]. Loop-mediated isothermal amplification (LAMP), one notable alternative, has garnered acclaim for its ability to amplify DNA rapidly and efficiently under isothermal constant temperature conditions, eliminating the need for a thermal cycler. This feature makes LAMP particularly suitable for resource-constrained environments [29]. Similar to LAMP, SYBR Green technology offers several advantages. One of the primary advantages is its cost-effectiveness and simplicity. SYBR Green technology relies on the interaction of a fluorescent dye with dsDNA, in contrast to TaqMan technology, which depends on the exonuclease activity of the Taq polymerase enzyme and dual-labeled oligonucleotides, potentially incurring higher costs, as indicated by Mahmoudian et al. [30]. This simplicity and cost-effectiveness make SYBR Green-based real-time PCR a practical choice for pathogen detection, especially in resource-constrained environments, akin to the attributes that make LAMP suitable for such settings.

The existing scientific literature lacks any documentation of a diagnostic methodology for CHV1 involving LAMP and SYBR Green-based real-time PCR, which serves as the central focus of this research. In this study, two distinct diagnostic protocols have been introduced, meticulously designed for precise CHV1 detection. These protocols utilize LAMP and qPCR techniques employing SYBR Green chemistry. Significantly, the methodologies incorporate innovative assay designs engineered to expedite and enhance the accuracy of CHV1 diagnosis in infected samples. The primary objective is to promote the adoption of LAMP and real-time PCR techniques as specialized and portable tools for pathogen detection. This, in turn, is intended to facilitate their application in on-site CHV1 surveillance initiatives, particularly in resource-limited regions.

## 2. Materials and Methods

### 2.1. Source of CHV1

**Figure 1:**
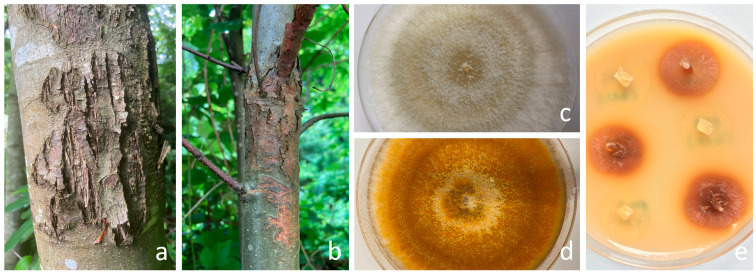
(**a**) Healed chestnut blight canker indicative of *Cryphonectria parasitica* hypovirulence. (**b**) Virulent chestnut blight canker. (**c**) Culture morphology of *C*. *parasitica* isolates: CHV1-infected YL-10 (white) and (**d**) CHV1-free EU-1 (orange) on PDA. (**e**) Bavendamm assay: brown coloration corresponds to polyphenol oxidase activity of the wild-type EU-1 isolates, while the weak color change in YL-10 suggests potential hypovirulence.

The 10 *Cryphonectria parasitica* isolates used in the study were obtained from only healing cankers collected from the Akçakoca location of the Bolu Forestry Management Directories (Figure 1a,b; Table 1). The YL-10 isolate (Figure 1c, GenBank accession number: OQ281817), known to contain CHV1 obtained from studies by Çakar [31], was employed to optimize experimental procedures in this study. The wild types of two European tester strains, EU-1 (Figure 1d) and EU-12, supplied by Dr. Daniel Rigling and Dr. Paolo Cortesi, were included in the studies as negative controls.

### 2.2. Bavendamm Test

Fungal isolation was carried out by placing samples from 10 collected cankers onto potato dextrose agar (PDA; Difco, Sparks, MD, USA). To qualitatively assess phenol oxidase (laccase) activity, the Bavendamm test was employed. This test involved cultivating the isolates on an agar medium containing tannic acid, known as Bavendamm medium, following the procedure outlined by Rigling et al. [32]. The plates were then placed in the dark at a temperature of 24 °C for four days, with additional observations made after 7 and 14 days. The phenol oxidase activity was visually recorded, and isolates displaying a weak color change were considered potentially hypovirulent. The Bavendamm test was conducted with three replications to ensure the reliability of the results.

### 2.3. RNA Extraction, DNase Treatment, and cDNA Synthesis

RNA extraction from CHV1-infected colonies and control groups was carried out using 50 mg of the sample in accordance with the manufacturer’s instructions. NucleoZOL RNA extraction solution (Macherey-Nagel GmbH & Co., Dueren, Germany) was used for this purpose. DNase digestion was performed using RNase-Free DNase (Thermo Fischer Scientific, Waltham, MA, USA) to remove any potential DNA contamination. The resulting 2 µg RNA was then subjected to cDNA synthesis using the RevertAid First Strand cDNA Synthesis Kit (Thermo Fischer Scientific), following the manufacturer’s protocols. The resulting cDNA samples were 10-fold diluted to 10 ng cDNA/μL and stored at −20 °C for subsequent quantification studies. To assess the quality and concentration of nucleic acids, absorbance measurements at 260 nm and 280 nm were conducted using a DS-11 FX+ series spectrophotometer (Denovix Inc., Wilmington, DE, USA).

### 2.4. Primer Design

**Figure 2:**
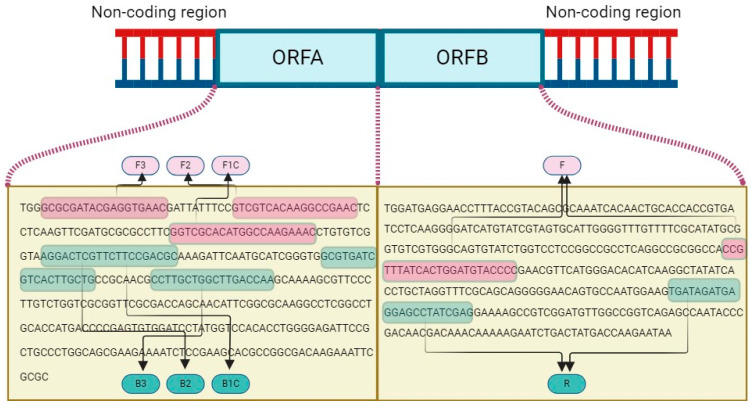
Schematic representation of LAMP (FIP primer consisting of F1C+F2 and BIP primer consisting of B1C+B2) and qPCR primer (F and R) binding sites on ORF A and ORF B of CHV1 genome.

By comprehensive alignment of the ORF A and ORF B regions of both Turkish and global 153 CHV1 isolates available in the GenBank database, a consensus segment was established 154 consisting of 362 (ORF A) and 9495 (ORF B) bp lengths. Subsequently, the most conserved region of the YL-10 was identified, designated, and used as input data for LAMP primer design by using the Primer Explorer V5 (PE5) tool (http://primerexplorer.jp/lampv5e/index.html, accessed on 15 May 2023) based on default parameters. Furthermore, for the development of qPCR primers, the viral isolate bearing the accession number KY471627 was chosen as the reference sequence for primer design, with a specific focus on the ORF B region (targeting between 4142 and 4260 nt on ORF B) by employing the NCBI primer design tool (https://www.ncbi.nlm.nih.gov/tools/primer-blast/, accessed on 15 May 2023). The primers were chosen manually, considering amplicon size, melting temperature, position, GC content, and annealing temperatures. The results were validated using the Beacon Designer (http://free.premierbiosoft.com, accessed on 15 May 2023) and mFold (http://www.idtdna.com/Scitools/Applications/mFold/, accessed on 15 May 2023) platforms. The new diagnostic primers (Table 2) were custom synthesized by Oligomer Biotechnology Inc. in Ankara, Türkiye, using HPLC purification. Additionally, the Basic Local Alignment Search Tool (BlastN) was utilized to verify primer specificity within the GenBank database. The list of designed oligonucleotides and schematic representation of the binding sites of the designed primers are provided in detail in Figure 2.

### 2.5. Initial Conditions of Colorimetric LAMP and SYBR Green qPCR

To detect CHV1 in the LAMP experiment, the WarmStart Colorimetric RT-LAMP 2× Master combination kit (NEB, Ipswich, MA, USA) was utilized. The reaction mixture consisted of inner [FIP (5′-F1C-F2-3′) and BIP (5′-B1C-B2-3′)] and outer (F3 and B3) primers (10 μM), 2× Colorimetric LAMP Master Mix (12.5 μL), and a cDNA template (10 ng/μL), resulting in a final volume of 25 μL. The colorimetric LAMP reaction was carried out at 65 °C for 30 min. Subsequently, the reaction was visually inspected and evaluated using agarose gel electrophoresis, and the results were documented by photography.

The qPCR analysis was performed in the CFX Connect Real-Time PCR System (Bio-Rad, Hercules, CA, USA) using a 25 μL reaction mixture. This mixture included 12.5 μL of RealQ Plus 2× Master Mix Green (Ampliqon, Odense, Denmark), 0.75 μL of each primer (F and R) (10 μM final), 1 μL of cDNA, and 10 μL of RNase-free ddH_2_O. The qPCR process was initiated with an initial cycle at 95 °C for 15 min to activate the TEMPase hot-start enzyme, followed by 40 cycles. Each cycle involved denaturation at 95 °C for 15 s, annealing at 61 °C for 45 s, and extension at 72 °C for 20 s. Following the completion of the PCR cycles, a melting curve analysis was conducted by measuring the fluorescence continuously when heating from 65 to 95 °C at the rate of 0.2 °C per second to distinguish specific and non-specific PCR products.

### 2.6. Sensitivity and Specificity of the LAMP and SYBR Green qPCR

To evaluate sensitivity, a six-fold cDNA dilution series from 10 ng to 100 fg cDNA/μL was utilized in the experiments for conventional RT-PCR, LAMP, and qPCR to detect the lower detection limit of each assay. The experiments incorporated the CHV1-free isolates (EU1 and EU12), the cDNA of *C*. *parasitica*, and RNase-free ddH_2_O as key components to ensure assay specificity. Furthermore, consultation with the NCBI online database was undertaken to evaluate potential unintended primer matches, mitigating the risk of false-positive results due to undesired primer binding. Two RT-PCR procedures were carried out using hvep1 and hvep2 primers for ORF A and 12F and 12R primers for ORF B according to protocols described by Gobbin et al. [20] and Feau et al. [33], respectively. These established procedures were employed as reference methods for comparison.

### 2.7. Comparison of the Assays

A total of ten CHV1 isolates were obtained from a previously conducted research study [31]. The isolates underwent comparison through the utilization of both colorimetric LAMP and qPCR methodologies. Furthermore, the comparison approach incorporated the inclusion of the well-established Bavendamm test, which has been traditionally utilized for the diagnosis of CHV1 [32]. This facilitated a thorough and rigorous comparative evaluation of the relative effectiveness and accuracy of different diagnostic methods.

## 3. Results

### 3.1. Optimization and Validation of Primer Sets for CHV1 Diagnosis Using LAMP and qPCR

**Figure 3:**
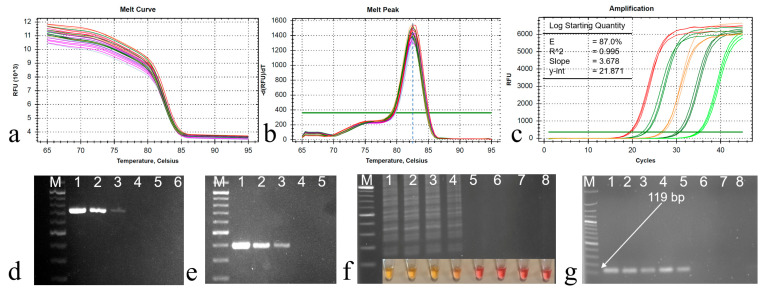
(**a**) Melting curve and (**b**) melting peak analysis of the qPCR products, a single melting peak at 82.5 °C, (**c**) amplification curve for sensitivity determination with 10-fold dilution series (DS) of the cDNA starting from 10 ng to 100 fg cDNA/µL (lines 1 to 6), (**d**) PCR products of DS for ORF B with 12F and 12R primers, (**e**) PCR products of DS for ORF A with hvep1 and hvep2 primers, (**f**) LAMP reaction products for DS, (**g**) qPCR amplifications of DS with the primer pairs CHV-F/CHV-R (119 bp). M: 100 bp DNA ladder (Solis BioDyne, Tartu, Estonia). Line 7: CHV1-free isolates (EU1), and Line 8: RNase-free ddH_2_O.

The qPCR primer sets exhibited the amplification of a specific product from the ORF B region of CHV1, as demonstrated by the well-defined melt curve in Figure 3a. No amplification was observed in the control groups, indicating the specificity of these primers. Importantly, no primer dimers were detected in the melting curve analysis. Upon analysis, the melting curve displayed a distinct peak at 82.5 °C when CHV1 cDNA was present, confirming the primers’ specificity (Figure 3b). This was further corroborated through agarose gel electrophoresis, which revealed the presence of the amplified 119 bp products.

The efficiency of the primer set was determined using the slopes from the standard curve via CFX Maestro Software 2.3 (Bio-Rad). The cDNA dilution series exhibited a high efficiency of 0.87 and exceptional linearity, with an R^2^ value exceeding 0.995 and a slope value of 3.678 (Figure 3c). Consequently, the precise annealing temperature (61 °C) for qPCR was established and subsequently applied in all subsequent reactions. Products of two RT-PCR procedures were also given in Figure 3d,e.

Four distinct primer sets were designed to target specific nucleotide sequences in the ORF A region, essential for CHV1 diagnosis using LAMP. However, only the primer set outlined in Table 2 demonstrated a clear color change, indicating positive amplification. It also produced a ladder-like pattern upon the initial reaction, confirming successful amplification. Subsequently, the optimization process focused exclusively on this specific primer set. Following the guidelines from the WarmStart Colorimetric RT-LAMP 2× Master kit, the initial reaction at 65 °C for 30 min yielded no observable amplification. Further experimentation was carried out to determine the appropriate reaction time, and it was found that a 35 min duration produced successful results when using a cDNA concentration of 10 ng/μL. After 35 min of incubation, the LAMP assay displayed a noticeable color change, transitioning from pink to yellow. This observed color transformation served as an indicator of the presence of CHV1 cDNA within the sample. Furthermore, distinct ladder-like banding patterns were clearly visible, providing conclusive confirmation of the successful amplicon of the target gene for LAMP (Figure 3f) and a 119 bp amplification for qPCR (Figure 3g).

### 3.2. Sensitivity and Specificity Assessment of Novel LAMP and qPCR Assays

Sensitivity testing included generating a 10-fold dilution series of cDNA (from 10 ng to 100 fg cDNA/µL) to evaluate the diagnostic performance of conventional PCRs and the newly developed LAMP and qPCR methods. Importantly, in comparison to two established classical PCR techniques that detected three dilution series (to 100 pg cDNA/µL), the LAMP method displayed a sensitivity 10 times higher (to 10 pg cDNA/µL), and the qPCR approach exhibited an impressive 100-fold increase in sensitivity, detecting five diluted cDNA (to 1 pg cDNA/µL). Quantification cycle (Cq) values for the dilution series were 19.52 (10 ng cDNA/µL), 23.14 (1 ng/µL), 26.74 (100 pg/µL), 30.47 (10 pg/µL), and 34.29 (1 pg/µL).

Regarding the specificity experiments for the two novel assays, the cDNA originating from the CHV1-free isolate (EU2), *C*. *parasitica*, and ddH_2_O all produced negative results, confirming the absence of false positives. Additionally, a thorough examination of the NCBI online database yielded no indications of potential undesirable primer matches, further affirming the specificity of the assay.

### 3.3. Comparative Analysis of the Methods

The results presented in Table 3 indicate that the molecular-based diagnostic techniques successfully identified all 10 samples. Interestingly, the Bavendamm assay yielded negative results for three of these samples. During the comparative analysis phase of this study, conventional PCR, LAMP, and qPCR methods clearly outperformed the Bavendamm test. The analysis of 10 isolates demonstrated a significantly higher level of sensitivity associated with these molecular-based methods. The Bavendamm test, assessing phenol oxidase (laccase) activity qualitatively, identified weak color change in seven out of ten isolates (Figure 1e). Notably, a strong correlation was observed between the Cq values and Bavendamm test results. Isolates with Cq values surpassing the test average of 28.44 consistently yielded negative Bavendamm results, indicative of hypovirulence.

## 4. Discussion

*Cryphonectria parasitica*, an indigenous fungal species originally native to Asian regions, notably China, Japan, and Korea, as documented by Liu et al. [7], has been recurrently introduced into diverse geographical areas, including Türkiye, primarily through chestnut plant trade and reforestation activities as reported by Akıllı et al. [34]. The challenges associated with eradicating *C*. *parasitica* via conventional methods, such as tree or branch removal followed by incineration, have been conspicuous in multiple regions, often yielding unsuccessful outcomes, as observed in various studies, including Rigling and Prospero [35]. Consequently, the growing interest in biocontrol approaches, particularly the natural infection of the fungus by CHV1, underscores the necessity for further exploration and implementation of alternative strategies for the effective management of chestnut tree blight. The insights and methodologies presented in this paper contribute novel perspectives to the diagnosis of CHV1, a pivotal element in implementing this biological control approach.

Upon an extensive literature review, it becomes evident that the diagnostic investigation of CHV1 has primarily relied on conventional PCR methodologies, as documented in studies by Grimaldi et al. [18], Allemann et al. [19], and Gobbin et al. [20]. Furthermore, contemporary research reaffirms the continued effectiveness and relevance of PCR-based diagnostic approaches for accurate CHV1 detection, as evidenced by Chun et al. [25], Romon-Ochoa et al. [26], Romon-Ochoa et al. [27], and Romon-Ochoa et al. [36]. Nevertheless, the absence of a diagnostic protocol for CHV1 utilizing the LAMP and SYBR Green-based approaches within the existing literature highlights a significant knowledge gap. This study addresses this gap by introducing two novel diagnostic methods, thus contributing valuable advancements to the field of CHV1 diagnosis.

The effectiveness and quality of RNA and DNA amplification-based methodologies are contingent upon the meticulous design of primers and probes, as emphasized by Çelik et al. [37]. In the context of our experimental investigation, we strategically focused on the design of novel primers for LAMP and qPCR methods with the specific intent of targeting the ORF A and ORF B genes within the CHV1 genome, respectively. This deliberate selection was rooted in the extensive exploration of these genetic loci in prior studies, including those by Jacob-Wilk et al. [14], Krstin et al. [16], Griffin et al. [21], Sotirovski et al. [22], Castaño et al. [23], and Rigling et al. [24]. Investigations into the genetic diversity of CHV1 have revealed a notable propensity for mutation and polymorphism, as indicated in studies by Gobbin et al. [20] and Nuskern et al. [38]. These findings align with the challenges encountered in identifying a conserved region suitable for primer design in our study. Consequently, the YLV-10 and KY471627 isolates were deliberately selected for primer design and subsequently applied in the empirical comparison of both methodologies. The newly crafted primers have conclusively demonstrated their efficacy in the detection of CHV1, thereby comparing the suitability of these approaches.

Numerous online tools have emerged in the realm of primer design, catering to diverse preferences and accessibility needs. One such tool, PE5, developed by Eiken Chemical Co., has gained widespread adoption for LAMP applications, proving to be a popular choice in numerous research endeavors. In contrast, tools like LAVA (LAMP Assay Versatile Analysis) and LAMP Designer (Optigene, Horsham, England) offer advanced functionalities but require a licensing fee, as pointed out by Torres et al. [39]. Thanks to its open accessibility, PE5 stands out as a favorable option for LAMP primer design. A similar scenario unfolds in the domain of qPCR primer design, where the NCBI online primer design tool demonstrates practicality and cost-effectiveness, aligning with the research conducted by Alkan et al. [40] and Çelik et al. [41].

The LAMP technique, initially introduced by Notomi et al. [42], is a DNA amplification method renowned for its capacity to rapidly detect nucleic acids from various sources, including viruses, bacteria, and plants. This method operates within a narrow temperature range of 60–65 °C, employing a minimal set of two to three complementary primers. LAMP’s simplicity allows for execution using basic equipment, such as a block incubator, as highlighted by Kurosaki et al. [43] and Kitamura et al. [44]. The visualization of LAMP results often entails the use of an indicator dye, leveraging the isothermal amplification process for straightforward observation, as demonstrated by Wang et al. [45]. Various dyes, including hydroxy naphthol blue [46], metal-sensitive indicators like calcein [47], the Warmstart 2× colorimetric LAMP kit [48], and SYBR Green I [49], can be employed to facilitate a visible LAMP reaction. The validation of the amplified target nucleic acid can be achieved through UV light observation and agarose gel electrophoresis, following the method described by Pham et al. [50].

In contrast to PCR-based methods, whether conventional or real-time, which typically necessitate two hours or more for completion, previous research consistently indicates that LAMP produces results within an average timeframe of 35 to 50 min, as noted by Notomi et al. [49] and Stehlíková et al. [51]. In our study, a successful colorimetric LAMP assay was developed using the Warmstart 2× colorimetric LAMP kit to identify CHV1 at 65 °C for 35 min. This outcome aligns with previous findings in LAMP studies, as documented by Golabi et al. [52] and Peltzer et al. [53]. It is worth noting that LAMP is associated with relatively higher costs compared to traditional PCR, primarily due to the requirement for additional primers and costly enzymes. However, it can be executed in a more cost-effective and straightforward manner using a heat block, rendering LAMP an optimal choice directly in field surveys and in scenarios where a significant initial investment is impractical or undesirable, as proposed by Çelik [29].

In recent years, a substantial body of research has been devoted to the design, development, and validation of qPCR techniques, as documented by Olveria et al. [54]. Previous investigations have extensively explored the application of probe-based real-time PCR for the diagnosis and quantification of CHV1, exemplified in studies by Chun et al. [25] and Romon-Ochoa et al. [26]. However, it is worth emphasizing that, up to the present time, a conspicuous dearth of the literature exists documenting any diagnostic approach employing SYBR Green-based real-time PCR, a focal point of this paper. This study introduces a novel SYBR Green-based qPCR method for the detection of CHV1. This newly developed qPCR assay presents numerous advantages over conventional PCR methods, encompassing heightened sensitivity, specificity, and the capacity for quantitative measurements. Importantly, the outcomes of this assay are congruent with those of previous qPCR methodologies developed for a variety of other plant viruses, as evidenced in studies by Mumford et al. [55], Sharma and Dasgupta [56], Herrera-Vásquez et al. [57], and MacKenzie et al. [58].

The selection of SYBR Green technology is underpinned by its simplicity and cost-effectiveness, relying on the interaction of the fluorescent dye with dsDNA, in contrast to the TaqMan technology, which is contingent upon the exonuclease activity of the Taq polymerase enzyme and dual-labeled oligonucleotides, incurring higher costs, as elucidated by Mahmoudian et al. [30]. The chosen primer set has demonstrated remarkable efficiency in amplifying the target gene, affirming that SYBR Green-based qPCR represents a swift and sensitive approach for the direct detection of CHV1. Notably, our newly designed primers, targeting the ORF B gene region of CHV1, have exhibited effective detection capabilities with favorable efficiency (E), coefficient of determination (R^2^), and slope values, aligning with established standards, as established by Broeders et al. [59]. The possibility of false positive results due to SYBR Green’s intercalation into all double-stranded DNA is an important consideration in nucleic acid amplification assays. Performing melting curve analysis at the end of the amplification reaction of CHV1 detection can help distinguish specific amplicons from non-specific products based on their melting temperature. These results underscore the precision and linear response of the experiments across a wide spectrum of dilutions and suggest the absence of PCR inhibitors.

The accuracy of diagnosing a specific ailment crucially depends on the precision of target detection methods. In our investigation, diluted CHV1 samples underwent comprehensive analysis through LAMP, RT-PCR, and qPCR assays. Through sensitivity assessment, accomplished by generating a 10-fold dilution series, the exceptional diagnostic performance of the newly introduced LAMP and qPCR methodologies has been prominently highlighted. Notably, the LAMP methodology demonstrated a sensitivity ten times higher than RT-PCR, while the qPCR method exhibited an impressive 100-fold enhancement in sensitivity.

Previous investigations have explored the comparative sensitivities of LAMP and RT-PCR, revealing varying degrees of superiority for each technique, as evidenced by Notomi et al. [42] and Kokane et al. [60]. Importantly, our study reaffirms the compatibility of the quantity of nucleic acid used in RT-PCR with the requisites of the novel LAMP and qPCR assays for CHV1 identification, thereby underscoring the effectiveness of LAMP and qPCR as potent diagnostic tools.

To compare the performance of diagnostic methods, ten CHV1-infected samples were subjected to an array of diagnostic techniques, including the Bavendamm test, RT-PCR, LAMP, and qPCR. These assessments revealed that the three molecular-based diagnostic methodologies consistently outperformed the Bavendamm method in effectively identifying infected samples. Conversely, isolates with lower Cq values maintained phenol oxidase activity, signifying virulence. This robustly illustrates the connection between Cq values and hypovirulence in *C. parasitica*. These findings emphasize the potential of integrating molecular diagnostics, like LAMP and SYBR Green-based real-time PCR, with traditional phenotypic assessments like the Bavendamm test to enhance CHV1 diagnosis accuracy and hypovirulent strain identification.

Several studies, including those conducted by Fan et al. [61], Galvez et al. [62], and Rizzo et al. [63], have consistently emphasized the enhanced diagnostic capabilities of LAMP and qPCR in nucleic acid analysis. Simultaneously, serological-based diagnostic approaches for virus diagnosis appear notably less robust when compared to their molecular-based counterparts, as articulated by Candresse et al. [64]. These findings align with the outcomes of the Bavendamm test, a non-molecular-based diagnostic assay, characterized by reduced sensitivity and dependence on visible symptoms, contrasting sharply with the high sensitivity and specificity of PCR techniques. Consequently, PCR methods are proposed as conferring increased accuracy and reliability in CHV1 detection.

The choice of the appropriate diagnostic method hinges on the specific context, necessitating an evaluation of factors such as desired sensitivity, cost considerations, and resource availability. The results stemming from this research align with earlier findings, thus reinforcing the notion that both LAMP and qPCR assays exhibit remarkable efficacy in the detection of CHV1. Future research will focus on testing these two newly developed methods without the need of performing RNA extractions (toothpick colony reactions) and on their accuracy in combination in a pipeline with other pre-existing molecular techniques that permit subtyping and haplotyping the CHV1.

## 5. Conclusions

In conclusion, this study introduced and compared novel diagnostic approaches, namely LAMP and SYBR Green-based qPCR, for the precise diagnosis of CHV1. Comparative assessments were employed to evaluate the performance of these novel techniques in relation to established diagnostic methods, shedding light on their efficacy and potential. The findings from this investigation strongly indicate that these pioneer diagnostic methods hold significant promise for the detection of CHV1, particularly in regions with limited access to advanced laboratory infrastructure. The qPCR method exhibited remarkable resilience in diagnosing CHV1, even at low pathogen concentrations. In a broader context, the integration of these two innovative diagnostic techniques is poised to yield substantial contributions to the enhancement of CHV1 surveillance efforts and provide valuable insights into the biological control of *C*. *parasitica*.

## Figures and Tables

**Table 1:** Collection sites and characteristics of *Cryphonectria parasitica* samples.

Provinces/ Location	Coordinates	Isolates ID	Culture Color	Color of Bavendamm Test
Bolu/ Akçakoca	41.010615 N 31.182617 E	B_01	Cream	Weak
	41.010344 N 31.181339 E	B_02	Cream	Weak
	41.010182 N 31.181543 E	B_03	Cream	Weak
	41.009988 N 31.182508 E	B_04	Cream	Intensive
	41.010036 N 31.181747 E	B_05	White	Intensive
	41.013886 N 31.153606 E	B_06	Cream	Intensive
	41.013494 N 31.153015 E	B_07	White	Weak
	41.013446 N 31.153358 E	B_08	White	Weak
	41.013616 N 31.153229 E	B_09	Cream	Weak
	41.013340 N 31.154034 E	B_10	White	Weak
Yalova/ Esenköy	40.624436 N 28.995506 E	YL-10	supplied by Çakar [31]	
European tester strains	-	EU-1	supplied by Dr. Daniel Rigling and Dr. Paolo Cortesi	
	-	EU-2		

**Table 2:** The sequences of designed primers for both assays.

Assay	Target	Primer	Sequence (5′–3′)	Length (bp)
**LAMP**	ORF A	F3	GCGCGATACGAGGTGAAC	18
		B3	TTGGTCAAGCCAGCAAGG	18
		FIP	GTTTCTTGGCCATGTGCGACCGTCGTCACAAGGCCGAAC	39
		BIP	AAGGACTCGTTCTTCCGACGCCAGCAAGTGACGATCACGC	40
**qPCR**	ORF B	F	CCGTTTATCACTGGATGTACCC	22
		R	TGATAGATGAGGAGCCTATCGAG	23

**Table 3:** Comparative analysis of four methods for CHV1 detection.

Samples	Bavendamm	PCR	LAMP	qPCR
1	+	+	+	+
2	+	+	+	+
3	-	+	+	+
4	+	+	+	+
5	+	+	+	+
6	-	+	+	+
7	+	+	+	+
8	+	+	+	+
9	-	+	+	+
10	+	+	+	+
EU1	-	-	-	-
EU12	-	-	-	-
ddH_2_O	-	-	-	-

## Data Availability

The datasets generated and analyzed during the current study are available upon reasonable request.
